# *Chlamydia trachomatis* and the Risk of Pelvic Inflammatory Disease, Ectopic Pregnancy, and Female Infertility: A Retrospective Cohort Study Among Primary Care Patients

**DOI:** 10.1093/cid/ciz429

**Published:** 2019-08-24

**Authors:** Casper D J den Heijer, Christian J P A Hoebe, Johanna H M Driessen, Petra Wolffs, Ingrid V F van den Broek, Bernice M Hoenderboom, Rachael Williams, Frank de Vries, Nicole H T M Dukers-Muijrers

**Affiliations:** 1 Division of Pharmacoepidemiology and Clinical Pharmacology, Utrecht Institute of Pharmaceutical Sciences, Heerlen; 2 Department of Sexual Health, Infectious Diseases and Environmental Health, Public Health Service South Limburg, Heerlen; 3 Department of Medical Microbiology,Care and Public Health Research Institute (CAPHRI), Maastricht University Medical Center+ (MUMC+), Maastricht; 4 Department of Clinical Pharmacy and Toxicology CAPHRI, School for Nutrition and Translational Research in Metabolism, MUMC+, Maastricht; 5 Epidemiology and Surveillance Unit, Centre for Infectious Disease Control, National Institute for Public Health and the Environment, Bilthoven, The Netherlands; 6 Clinical Practice Research Datalink, Medicines and Healthcare Products Regulatory Agency, London, United Kingdom

**Keywords:** *Chlamydia trachomatis*, adverse reproductive health, CPRD, antibiotics

## Abstract

**Background:**

We evaluated the risk of pelvic inflammatory disease (PID), ectopic pregnancy, and infertility in women with a previous *Chlamydia trachomatis* (CT) diagnosis compared with women who tested negative for CT and CT untested women, considering both targeted and incidental (ie, prescribed for another indication) use of CT-effective antibiotics.

**Methods:**

This was a retrospective study of women aged 12–25 years at start of follow-up within the Clinical Practice Research Datalink GOLD database linked to index of multiple deprivation quintiles, 2000–2013. CT test status and antibiotic use were determined in a time-dependent manner. Risk of PID, ectopic pregnancy, or female infertility were evaluated using of Cox proportional hazard models.

**Results:**

We studied 857 324 women, contributing 6 457 060 person-years. Compared with women who tested CT-negative, women who tested CT-positive had an increased risk of PID (adjusted hazard ratio [aHR], 2.36; 95% confidence interval [CI], 2.01–2.79), ectopic pregnancy (aHR, 1.87; 95% CI, 1.38–2.54), and infertility (aHR, 1.85; 95% CI, 1.27–2.68). The PID risk was higher for women with 2 or more positive CT tests than those with 1 positive test. PID risk increased with the number of previous antibiotic prescriptions, regardless of CT test status.

**Conclusions:**

We showed an association between CT-positive tests and 3 adverse reproductive health outcomes. Moreover, this risk increased with repeat CT infections. CT-effective antibiotic use showed no decreased risks of subsequent PID regardless of CT history. Our results confirm the reproductive health burden of CT, which requires adequate public health interventions.

## Teaser

Time-dependent analyses on a dataset including 6.5 million person-years confirms a strong relationship between *Chlamydia trachomatis* (CT) and pelvic inflammatory disease (PID), ectopic pregnancy, and female infertility. CT-effective antibiotic use showed no decreased PID risk in CT untested women.

**(See the Brief report by Reekie et al on pages 1621–3.)**

Chlamydia is a sexually transmitted infection caused by the bacterium *Chlamydia trachomatis* (CT) and is characterized by a high proportion of asymptomatic infections (up to 70% in women) [1]. In women, CT can ascend from the lower genital tract and thereby affect the uterus, fallopian tubes, and ovaries, resulting in pelvic inflammatory disease (PID) [2]. Subsequently, PID can lead to several adverse reproductive health outcomes, including ectopic pregnancy and infertility. In this respect, several studies have reported on associations between CT and adverse reproductive health outcomes [3–5].

However, the strength of the association between CT and adverse reproductive health outcomes is part of an ongoing scientific debate. In a large Danish retrospective cohort study, Davies et al observed that the risk of developing PID, ectopic pregnancy, or tubal factor infertility was at least 30% higher in women with 1 or more CT-positive tests compared with women with only CT-negative tests [4]. In another cohort study that included 120 000 Western Australian women, a higher PID risk (80%) was observed among women who tested CT-positive compared with those who tested CT- (and gonorrhea) negative [5]. In a UK modeling study, Price et al estimated 171 cases of PID, 73 cases of salpingitis, and 2 cases of ectopic pregnancy for every 1000 women with untreated CT [6]. So, although the exact risk of reproductive complications after chlamydia infection is estimated differently across studies, chlamydial PID remains the most important preventable cause of infertility and adverse reproductive health outcomes [7, 8].

There is consensus about the importance of early antenatal detection and effective CT treatment [9, 10]. CT-effective antibiotics prescribed for another bacterial infection can additionally treat asymptomatic CT. As such, CT can be treated “incidentally” [2], which has been shown in an Australian study and a study from our research group [11, 12]. However, the incidental beneficial effects of antibiotics on the association between CT and adverse reproductive health outcomes have not been evaluated.

For this reason, our objective in this study was to assess the incidence of PID, ectopic pregnancy, and female infertility in women with a previous CT diagnosis compared with women who have tested negative for CT and women who have not been tested for CT. We also evaluated the impact of antibiotic use on our outcomes of interest.

## METHODS

### Data Sources

We conducted a retrospective cohort study using the Clinical Practice Research Datalink (CPRD) GOLD. CPRD GOLD collates the computerized medical records for more than 11 million patients from 674 general practices in the United Kingdom, which are representative of the UK population [13]. In this way, this work uses data provided by patients and collected by the National Health Service as part of the patients’ care and support.

### Study Population

**Figure 1.**
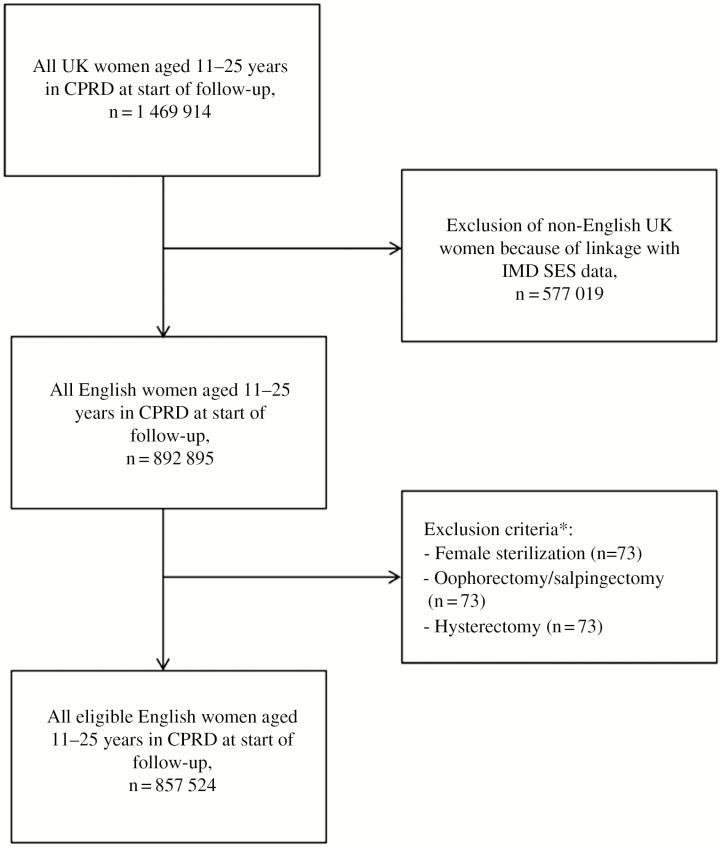
Flow diagram of women included in the study per exclusion step. Abbreviations: CPRD, Clinical Practice Research Datalink; IMD, index of multiple deprivation; SES, socioeconomic status; UK, United Kingdom. *Numbers add up to more than the total number of women excluded in this step because a woman could have a history of more than 1 of the excluded conditions.

Included at the start of follow-up were all women aged between 12 and 25 years, regardless of a history of previous CT infections [14]. Our CPRD GOLD dataset was linked to the 2010 English index of multiple deprivation (IMD) in order to include socioeconomic status (SES) in our analyses. In this way, only women living in England were included in the dataset. Women with a history of hysterectomy, bilateral oophorectomy/ovariectomy, or sterilization (eg, tubal ligation) were excluded from the cohort (Figure 1). For each woman, the period of valid data collection started at 1 January 2000, 31 December of the year a woman became 11 years old, the date on which the practice became “up to research standard” according to CPRD data quality criteria, or the date that the practice started contributing data to CPRD GOLD, whichever came latest.

### Exposure

**Figure 2.**
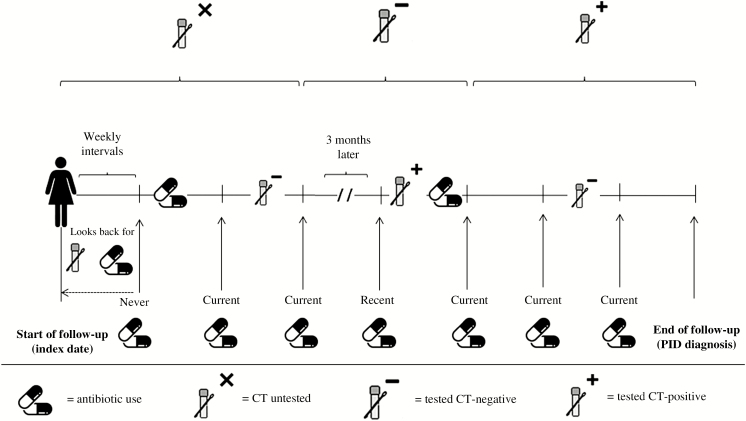
Classification of follow-up time according to CT test status and antibiotic use. Abbreviations: CT, *Chlamydia trachomatis*; PID, pelvic inflammatory disease. Never use = no antibiotic prescription was issued during follow-up; current use = the most recently recorded antibiotic prescription was issued in the past 3 months; recent use = the most recently recorded prescription was issued between 4 months and 3 years ago.

Patient follow-up time was divided into weekly intervals at which exposure status (ie, CT testing and CT-effective antibiotic use) for each woman was assessed following the methodology given below and illustrated in Figure 2.

#### Chlamydia Testing Status

Cohort entry defined the start of follow-up (ie, the index date). During follow-up, testing status was categorized into 3 hierarchical groups: CT untested, CT negative, and CT positive. CT infections were diagnosed in England using routine practices, that is, serology (until 2006) and nucleic acid amplification tests. A person in the dataset could move unidirectional to the next category during follow-up (but could not go back) [15]. In case a test was not followed by a diagnosis, we assumed that the test had a negative result. The date of CT diagnosis was defined as the moment of the CT diagnosis according to medical codes in the CPRD GOLD database or of the CT treatment event (product codes) within 2 weeks from CT diagnosis (CPRD codes available in the Supplementary Materials).

To evaluate the influence of repeat infections, the exposure group and corresponding follow-up time of CT-positives were hierarchically divided by the number of positive tests (1, 2, more than 2). A time window of 3 months was required between 2 positive tests to be considered indicative of a repeat infection in order to avoid misclassification by counting the same infection multiple times.

#### Antibiotic Use

The following CT-effective antibiotics, including the minimum dosage for antibiotics listed in the British Association for Sexual Health and HIV guidelines, were included: azithromycin (250 mg), doxycycline (50 mg), ofloxacin (200 mg), erythromycin (250 mg), amoxicillin (250 mg), clindamycin, clarithromycin, tetracyclines (other than doxycycline), and penicillins (other than amoxicillin) [16–18]. Minimum dosages were included because of doubt of significant activity against CT below these thresholds.

Treatment was evaluated at the start of each interval. Until an antibiotic was prescribed, intervals were classified as “never use.” Hereafter, based on the time since the most recent prescription, antibiotic use was classified per interval as “current use” (the most recently recorded prescription was issued in the past 3 months), “recent use” (issued in the past 4 months to 3 years), or “past use” (issued more than 3 years ago).

In order to evaluate the effect of CT-effective treatment on the outcomes in more detail, follow-up time for recent antibiotic use was stratified by the number of prior antibiotic prescriptions (1, 2–3, and more than 3). This was done for recent antibiotic use and not for current use in order to limit reverse causality (treatment related to outcome) and to ensure that most women could contribute to this follow-up time evaluated in more detail.

### Follow-up Time

Women were followed up from the index date to the end of data collection (31 December 2013), the date of transfer of the patient out of the practice area, the patient’s death, or an episode of the outcome of interest (ie, PID, ectopic pregnancy, or female infertility, based on CPRD medical codes; see the Supplementary Materials), whichever came first. For each individual outcome, a separate analysis was conducted.

### Potential Confounders

Demographic risk factors were considered as potential confounders in the evaluation of all associations. In this respect, smoking, body mass index, and SES (CPRD provided the linked quintiles based on the 2010 English IMD) were assessed at study baseline. Missing data on demographic information were grouped in a separate category per variable and included in the analyses. Age, handled as a continuous variable, and all other confounders were reviewed in a time-dependent manner, that is, at the start of each time interval.

In addition, analyses for PID and ectopic pregnancy were adjusted for a history of gonorrhea. Female infertility analyses were adjusted for a history of gonorrhea, PID, amenorrhea, previous pregnancy, and the use of oral contraceptives in the previous 6 months. The full list of evaluated confounders is given in the Supplementary Materials.

### Data Analyses

Incidence rates were calculated as events per 1000 person-years. We used Cox proportional hazards regression models to estimate the associations between exposure groups with the hazard of developing each outcome, controlling for potential confounders (using Stata Statistical Software, release 14, StataCorp LP, College Station, TX).

Specifically, we calculated the adjusted hazard ratio (aHR) for CT-positive and CT-untested women vs CT-negative women (reference group); in a subanalysis, the CT-positive women were further stratified by the number of positive tests. In addition, we calculated aHR for all CT-effective antibiotic use categories stratified for CT testing status vs CT-negative women without antibiotic use (reference group), in which recent users were further stratified by number of antibiotics used.

Sensitivity analyses are given in the Supplementary Materials (Supplementary Tables 1–3), which did not change results significantly.

## RESULTS

We identified 857 324 women who had a mean duration of follow-up of 7.5 years, thereby contributing 6 457 060 person-years. The baseline characteristics of the study population are given in Table 1. The total numbers per outcome of interest were: 8346, PID; 2484, ectopic pregnancy; and 2066, female infertility. Mean age at baseline was 15 years, and a history of gonorrhea was rarely reported at study start.

**Table 1.** Baseline characteristics of the included women at start of follow-up

Characteristic	N	%
Mean duration of follow-up for PID (years, SD)^a^	7.5	4.3
Mean age in years(SD)	15	4.4
Mean BMI in kg/m^2^(SD)	23.3	5.6
Missing	272116	31.7
Smoking status		
Current	221,333	25.8
Ex	45,685	5.3
Never	426,763	49.8
Missing	163,543	19.1
SES		
Low	116,228	13.6
Medium-low	185,094	21.6
Medium	180,095	21.0
Medium-high	187,422	21.9
High	188,485	22.0
Disease history		
Gonorrhoea	110	0.01
Polycystic ovary syndrome	1,937	0.2
Hyperprolactinemia	63	0.01
Hypothyroidism	1,881	0.2
Hyperthyroidism	599	0.07
Endometriosis	530	0.06
Amenorrhea	5,755	0.7
Drug use 6 months before start of follow-up		
Folic acid	2,569	0.3
Immunosuppressants excl. corticosteroids	459	0.05
Oral contraceptives	49,211	5.7

A total of 857 324 women were included at start of follow-up.

^a^Values are numbers (percentages) unless stated otherwise.Abbreviations: BMI, body mass index; PID, pelvic inflammatory disease; SD, standard deviation; SES, social economic status.

### Risk in Outcomes

Incidence rates (per 1000 person-years) of PID were 1.1 (95% confidence interval [CI], 1.1–1.1) among women untested for CT, 1.4 (95% CI, 1.2–1.6) for CT-negatives, and 5.4 (95% CI, 4.9–6.0) for CT-positives. For ectopic pregnancy, these incidence rates were 0.3 (95% CI, 0.3–0.3) for untested, 0.4 (95% CI, 0.3–0.5) for negatively tested, and 1.2 (95% CI, 1.0–1.5) for positively tested women. Finally, infertility incidence rates were 0.3 (95% CI, 0.3–0.3) for untested, 0.3 (95% CI, 0.2–0.4) for CT-negatives, and 0.9 (95% CI, 0.7–1.1) for CT-positives.

Compared with CT-negatives, CT-positive women had an increased risk of PID (aHR, 2.36; 95% CI, 2.01–2.79), ectopic pregnancy (aHR, 1.87; 95% CI, 1.38–2.54), and female infertility (aHR, 1.85; 95% CI, 1.27–2.68; Table 2). In contrast, CT-untested women had lower risks compared with CT-negative women for PID (aHR, 0.57; 95% CI, 0.50–0.65).

**Table 2.** Risk for Pelvic Inflammatory Disease, Ectopic Pregnancy, and Female Infertility per *Chlamydia trachomatis* Test Status

Outcome by CT Test Status	Number of Outcomes per Category	Incidence Rate (per 1000 Person-years) (95% CI)	Age-adjusted HR (95% CI)	Adjusted HR (95% CI)
Pelvic inflammatory disease	8346	...	…	…
CT untested	7733	1.1 (1.1–1.1)	0.58 (.51–.66)	0.57 (.50–.65)^a^
CT negatives	235	1.4 (1.2–1.6)	Reference	Reference
CT positives	378	5.4 (4.9–6.0)	2.83 (2.40–3.32)	2.36 (2.01–2.79)^a^
Ectopic pregnancy	2484	…	…	…
CT untested	2320	0.3 (.3–.3)	0.85 (.67–1.07)	0.85 (.68–1.07)^a^
CT negatives	73	0.4 (.3–.5)	Reference	Reference
CT positives	91	1.2 (1.0–1.5)	2.12 (1.56–2.87)	1.87 (1.38–2.54)^a^
Female infertility	2066	…	…	…
CT untested	1952	0.3 (.3–.3)	1.05 (.79–1.40)	0.88 (.66–1.17)^b^
CT negatives	49	0.3 (.2–.4)	Reference	Reference
CT positives	65	0.9 (.7–1.1)	2.13 (1.47–3.09)	1.85 (1.27–2.68)^b^

Abbreviations: CI, confidence interval; CT, *Chlamydia trachomatis*; HR, hazard ratio.

^a^Adjusted for age, smoking status, and history of gonorrhea.

^b^Adjusted for age, smoking status, socioeconomic status, history of gonorrhea, amenorrhea, previous pregnancy, and use of oral contraceptives in the previous 6 months.

### Influence of Repeat Infections

The risk of PID was higher for women with 2 or more positive tests compared with 1 positive test (Table 3). For ectopic pregnancy, risk estimates were in similar directions, although not statistically significant; for female infertility, no such associations were observed (Table 3). However, it should be noted that the number of women who tested CT-negative or CT-positive with ectopic pregnancy (n = 164) or female infertility (n = 114) in these analyses was low.

**Table 3.** Associations Between Pelvic Inflammatory Disease, Ectopic Pregnancy, and Infertility and *Chlamydia trachomatis* Test Status by the Number of Tests

Outcome	CT Test Status by Number of Tests	Number of Outcomes per Category	Incidence Rate (per 1000 Person-years) (95% CI)	Age-adjusted HR (95% CI)	Adjusted HR (95% CI)
Pelvic inflammatory disease (n = 8346)	CT untested	7733	1.1 (1.1–1.1)	0.73 (.30–1.74)	0.70 (.29–1.67)^a^
	CT negatives by number of negative tests				
	1	192	1.4 (1.2–1.6)	1.22 (.50–2.95)	1.18 (.48–2.86)^a^
	2	38	1.6 (1.1–2.1)	1.54 (.61–3.91)	1.50 (.59–3.81)^a^
	≥3	5	1.0 (.1–1.8)	Reference	Reference
	CT positives by number of positive tests				
	1	320	5.2 (4.6–5.7)	3.37 (1.39–8.15)	2.76 (1.14–6.68)^a^
	2	48	7.6 (5.4–9.8)	4.83 (1.92–12.15)	3.69 (1.47–9.27)^a^
	≥3	10	7.8 (2.9–12.7)	5.24 (1.79–15.33)	3.78 (1.29–11.06)^a^
Ectopic pregnancy (n = 2484)	CT untested	2320	0.3 (.3–.3)	1.80 (.25–12.79)	1.77 (.25–12.55)^a^
	CT negatives by number of negative tests				
	1	62	0.4 (.3–.6)	2.04 (.28–14.72)	1.99 (.28–14.34)^a^
	2	10	0.4 (.2–.8)	2.11 (.27–16.50)	2.06 (.26–16.11)^a^
	≥3	1	0.2 (.0–1.4)	Reference	Reference
	CT positives by number of positive tests				
	1	77	1.2 (1.0–1.5)	4.40 (.61–31.66)	3.83 (.53–27.54)^a^
	2	12	1.8 (1.0–3.1)	6.24 (.81–47.99)	5.13 (.67–39.47)^a^
	≥3	2	1.4 (.3–5.6)	5.00 (.45–55.14)	3.95 (.36–43.61)^a^
Female infertility (n = 2066)	CT untested	1952	0.3 (.3–.3)	0.67 (.17–2.68)	0.42 (.10–1.68)^b^
	CT negatives by number of negative tests				
	1	37	0.3 (.2–.4)	0.57 (.14–2.36)	0.42 (.10–1.74)^b^
	2	10	0.4 (.2–.8)	1.05 (.23–4.78)	0.84 (.18–3.84)^b^
	≥3	2	0.4 (.1–1.5)	Reference	
	CT positives by number of positive tests				
	1	57	0.9 (.7–1.1)	1.36 (.33–5.56)	0.87 (.21–3.58)^b^
	2	7	1.0 (.5–2.2)	1.47 (.31–7.09)	1.03 (.21–4.95)^b^
	≥3	1	0.7 (.1–4.9)	1.04 (.09–1.15)	0.70 (.06–7.76)^b^

Abbreviations: CI, confidence interval; CT, *Chlamydia trachomatis*; HR, hazard ratio.

^a^Adjusted for age, smoking status, and history of gonorrhea.

^b^Adjusted for age, smoking status, socioeconomic status, history of gonorrhea, amenorrhea, previous pregnancy, and use of oral contraceptives in the previous 6 months.

### Antibiotic Use

For reasons of power, analyses on antibiotic use were performed only for the outcome PID. For all CT testing status, current antibiotic users had the highest risk of PID (Table 4). In addition, for recent users, the risk of PID increased with a higher number of CT-effective antibiotic prescriptions in the past.

**Table 4.** Associations Between Pelvic Inflammatory Disease and *Chlamydia trachomatis* Test Status by Antibiotic Use

CT Test Status by Antibiotic Use	Pelvic Inflammatory Disease (n = 8346)	Incidence Rate (per 1000 Person-years) (95% CI)	Age-adjusted HR (95% CI)	Adjusted HR^a^ (95% CI)
CT untested				
By effective antibiotic use				
Never^b^	2635	0.7 (.7–.8)	0.68 (.47–.97)	0.65 (.46–.94)
Ever	5098	1.4 (1.4–1.5)	1.29 (.90–1.85)	1.21 (.85–1.74)
By the time since the most recent antibiotic prescription				
Current^c^	1219	3.0 (2.8–3.1)	2.63 (1.83–3.77)	2.39 (1.66–3.43)
Recent^d^	3014	1.6 (1.6–1.7)	1.43 (1.00–2.05)	1.32 (.92–1.89)
Recent use by number of prescriptions^e^				
1 prescription	1227	1.3 (1.2–1.3)	1.13 (.79–1.62)	1.06 (.74–1.52)
2–3 prescriptions	1145	1.8 (1.7–1.9)	1.57 (1.09–2.26)	1.42 (.99–2.05)
≥4 prescriptions	642	2.6 (2.4–2.8)	2.29 (1.59–3.30)	2.06 (1.43–2.97)
Past^f^	865	0.7 (.6–.7)	0.65 (.45–.94)	0.64 (.45–.92)
CT negatives				
By effective antibiotic use				
Never^b^	30	0.9 (.5–1.2)	Reference	Reference
Ever	205	1.5 (1.3–1.8)	2.03 (1.38–2.97)	1.93 (1.32–2.83)
By the time since the most recent antibiotic prescription				
Current^c^	50	3.7 (2.7–4.8)	4.68 (2.98–7.36)	4.37 (2.78–6.87)
Recent^d^	119	1.7 (1.4–2.0)	2.13 (1.43–3.18)	2.00 (1.34–2.99)
Recent use by number of prescriptions^e^				
1 prescription	43	1.2 (.8–1.6)	1.54 (.97–2.46)	1.46 (.92–2.34)
2–3 prescriptions	50	1.9 (1.4–2.5)	2.51 (1.60–3.95)	2.34 (1.49–3.68)
≥4 prescriptions	26	2.6 (1.6–3.6)	3.35 (1.98–5.66)	3.13 (1.85–5.29)
Past^f^	36	0.8 (.5–1.0)	1.02 (.63–1.65)	0.99 (.61–1.62)
CT positives				
By effective antibiotic use				
Never^b^	68	6.4 (4.8–7.9)	5.50 (3.58–8.45)	4.20 (2.73–6.46)
Ever	310	5.3 (4.7–5.9)	4.99 (3.43–7.26)	4.08 (2.81–5.94)
By the time since the most recent antibiotic prescription				
Current^c^	104	10.3 (8.3–12.3)	9.94 (6.62–14.92)	8.15 (5.43–12.23)
Recent^d^	177	4.7 (4.0–5.4)	4.47 (3.04–6.59)	3.67 (2.49–5.40)
Recent use by number of prescriptions^e^				
1 prescription	55	3.8 (2.8–4.8)	3.54 (2.27–5.52)	2.92 (1.87–4.56)
2–3 prescriptions	66	4.3 (3.3–5.4)	4.19 (2.72–6.45)	3.42 (2.22–5.26)
≥4 prescriptions	56	7.0 (5.1–8.8)	7.03 (4.51–10.95)	5.72 (3.67–8.92)
Past^f^	29	2.7 (1.7–3.7)	2.45 (1.47–4.08)	2.02 (1.21–3.36)

Abbreviations: CI, confidence interval; CT, *Chlamydia trachomatis*; HR, hazard ratio.

^a^Adjusted for age, smoking status, and history of gonorrhea.

^b^Antibiotic use was classified per weekly follow-up interval as “never use” when no prescription was issued during follow-up.

^c^Antibiotic use was classified per weekly follow-up interval as “current use” when the most recently recorded prescription was issued in the past 3 months.

^d^Antibiotic use was classified per weekly follow-up interval as “recent use” when the most recently recorded prescription was issued between 4 months and 3 years ago.

^e^Stratification by the number of previous antibiotic prescriptions was only done for recent use and not for current use in order to avoid reverse causality (treatment related to outcome) and to ensure that most women could contribute to this follow-up time evaluated in more detail.

^f^Antibiotic use was classified per weekly follow-up interval as “past use” when the most recently recorded prescription was issued more than 3 years ago.

## DISCUSSION

In this large population-based retrospective cohort study, women who tested CT-positive had a substantially higher risk of experiencing PID (approximately 135%), ectopic pregnancy (approximately 90%), or female infertility (approximately 70%) than CT-negative women. An increasing number of positive CT tests (ie, repeat positive tests) was associated with a higher PID risk. In addition, CT-effective antibiotic use, in which targeted and incidental use were taken together, showed no decreased risks for PID regardless of CT testing status.

The strengths of this study were the large general practice cohort of more than 850 000 women taken from the CPRD GOLD database with “up-to-standard” data that are representative of the entire English population [13]. In this cohort, even the relatively rare outcomes, such as ectopic pregnancy, were available in sufficient numbers to evaluate the risk of our outcomes between the different CT testing categories. Moreover, exposure status and covariates were classified in a time-dependent manner, including a history of gonorrhea [4], which provides more accurate point estimates by preventing, for example, immortal time bias. Additionally, by censoring women’s follow-up time when the outcome of interest had occurred, we guaranteed that included (positive) CT tests were taken before the outcome, thereby preventing reverse causality. Finally, the effect of CT-effective antibiotic use on the risk of PID was comprehensively evaluated.

A limitation was that only data from primary care were available. In the United Kingdom, a sizeable proportion of sexually transmitted infections (STIs) are diagnosed and treated in genitourinary medicine clinics [19]. For this reason, there will be an underestimation of the number of CT diagnoses within our cohort, which could have diluted the effect of true associations. Even though we used the large CPRD GOLD dataset, we could determine the risk for ectopic pregnancy and female infertility only for the analysis between CT testing categories and not regarding the effect of the number of positive tests and antibiotic use because of insufficient power. Another limitation was that our outcomes were based on general practice (GP) records instead of hospital episodes. Although we expect many diagnoses from hospitals are received and registered by the GP, misclassification of the outcomes cannot be not ruled out. This is mainly applicable to PID, which has several associated signs and symptoms and no single diagnostic test available. However, the dose–response relationship observed between PID and the number of positive CT tests indicates a true association. Finally, our cohort was relatively young, with a mean age of 15 years at study start; therefore, a certain proportion would not have had the desire to become pregnant. This could have led to a potential underrepresentation of our outcomes, female infertility and ectopic pregnancy, among the younger women in our cohort. However, the results stratified by age group showed similar results across all strata.

Our findings are in line with those from a large Danish cohort study among women aged 15–44 years [4]. Davies et al showed at least a 30% higher risk of PID, ectopic pregnancy, and tubal factor infertility in women with 1 or more positive CT tests compared with women with only negative tests. Moreover, an 80% higher PID risk was shown for women who tested CT-positive compared with women who tested CT-negative in an Australian population-based cohort study [5]. The increased risk of these outcomes was more pronounced in our study, possibly because GP diagnoses of the outcomes were not included in the Danish cohort. In addition, we included female infertility in general as an outcome instead of the more specific tubal infertility outcome that was used in the Davies et al study.

Our observation that the risk for PID increased with an increasing number of positive CT tests is in accordance with previous studies. In the Danish cohort study by Davies et al, repeat CT infections resulted in an increase in PID risk by an additional 20% [4]. Similarly, a recent study by Bautista et al among army females in the United States showed a dose–response relationship between the number of CT diagnoses and the risk of PID [20]. Together with our data, these studies show that each (repeat) CT infection results in a higher risk of developing PID. These findings emphasize that CT screening should be targeted at high-risk groups and that diagnosis must be followed by optimal treatment in order to reduce the burden of CT.

With respect to the effect of incidental CT-effective antibiotic use, our group reported that recent non–CT-related tetracycline use was associated with a lower CT prevalence in a Dutch STI clinic population [11]. Moreover, Australian data showed that chlamydia positivity rates increased while national use of antibiotics effective against CT declined over a 10-year period [12]. These data imply that incidental treatment of CT infections may occur and, therefore, such treatment could have a reducing impact on the complications that follow a CT infection, that is, PID, ectopic pregnancy, and female infertility. On the contrary, CT-effective antibiotic use was associated with a higher PID risk in our study. Additionally, the PID risk was higher in women who had more than 1 CT-effective antibiotic prescription since the start of follow-up. This can be explained by the direct link between CT and PID for CT-positive women, but this relationship was also observed in CT-negative and untested women. With the effectiveness of CT treatment generally accepted, we do not consider it likely that antibiotic use in itself is a risk factor for PID.

Possible explanations for the positive association between PID and CT-effective antibiotic prescriptions could be that PID can be caused by other infectious diseases that could be treated with CT-effective antibiotics, for example, *Mycoplasma genitalium* with azithromycin as the treatment of first choice [21]. *Mycoplasma genitalium* has been detected in 13%–16% of women with PID [22]. Moreover, *Ureaplasma urealyticum* has been associated with infertility in serological studies [23]. In addition, it has been suggested that prior antibiotic use in STI clinic patients diagnosed with CT is related to earlier healthcare visits (eg, to the GP) with symptoms that in retrospect could be consistent with a CT infection, which could also be the case for PID [11]. In addition, the possible role of the immune system has been addressed regarding the progression of CT to PID [2]. Based on our findings, we can only speculate that a specific group of women is more vulnerable for (bacterial) infections due to immunological factors. Finally, the effect of antibiotics on the vaginal microbiome and the consequences on our outcomes need to be explored [24]. For this reason, more research is needed to evaluate which host and, specifically, which immunological factors could be involved in the progression of CT to PID and the possible implications of these findings on the susceptibility to infectious diseases in general. In addition, it would be interesting to know whether similar associations will be observed in countries with lower as well as higher antibiotic use than England in the general practice setting.

In conclusion, our findings provide evidence that women with a positive CT test registered at the GP have a higher risk for adverse reproductive health outcomes, irrespective of antibiotic use. Moreover, this risk increases with having more CT infections. Hence, our results confirm the reproductive health burden of CT and show the need for adequate public health interventions against CT to be in place.

## Supplementary Data

Supplementary materials are available at *Clinical Infectious Diseases* online. Consisting of data provided by the authors to benefit the reader, the posted materials are not copyedited and are the sole responsibility of the authors, so questions or comments should be addressed to the corresponding author.

ciz429_suppl_Supplementary_Files

## Notes

***Author contributions.*** C. J. P. A. H., F. de V., and N. H. T. M. D.-M. initiated the study, and C. D. J. den H. wrote the first draft of the paper. J. H. M. D. led the statistical analysis. All authors were responsible for the study concept and design and participated in the interpretation of data. All authors critically revised the manuscript for important intellectual content and approved the final version to be published. All authors had full access to all of the data (including statistical reports and tables) in the study and take responsibility for the integrity of the data and the accuracy of the data analysis. F. de V. supervised the study and is the guarantor.

***Acknowledgments.*** The authors thank all general practitioners and patients who contributed to the data presented here. This study was reviewed and approved by the independent scientific committee of the Clinical Research Practice Datalink (reference 15_144), which is responsible for reviewing protocols for scientific quality.

***Financial support.*** This study was supported by the Netherlands Organisation for Health Research and Development (ZonMW, project number: 522002013).

***Potential conflicts of interest.*** F. de V. supervises a doctorate student who is employed by F. Hoffmann, La Roche Ltd., Basel, Switzerland. The topic of his doctorate thesis is not related to this work. F. de V. has not received any fees or reimbursements for this. R. W. reports financial payments from Utrecht University to the Clinical Practice Research Datalink during the conduct of the study for access to study data and similarly from other organizations outside the submitted work. All other authors report no potential conflicts. All authors have submitted the ICMJE Form for Disclosure of Potential Conflicts of Interest. Conflicts that the editors consider relevant to the content of the manuscript have been disclosed.
