# Erratum to: *Chlamydia trachomatis* and the Risk of Pelvic Inflammatory Disease, Ectopic Pregnancy, and Female Infertility: A Retrospective Cohort Study Among Primary Care Patients

**DOI:** 10.1093/cid/ciz897

**Published:** 2019-09-19

**Authors:** Casper D J Den Heijer, Christian J P A Hoebe, Johanna H M Driessen, Petra Wolffs, Ingrid V F Van Den Broek, Bernice M Hoenderboom, Rachael Williams, Frank De Vries, Nicole H T M Dukers-Muijrers

An error appeared in the initial publication of this article [den Heijer CDJ, Hoebe CJPA, Driessen JHM, et al. Chlamydia trachomatis and the Risk of Pelvic Inflammatory Disease, Ectopic Pregnancy, and Female Infertility: A Retrospective Cohort Study Among Primary Care Patients. Clin Infect Dis https://doi.org/10.1093/cid/ciz429]. This article was originally published with the following error:

In the financial support section, it currently states the following:

“This study was supported by the Netherlands Organisation for Health Research and Development (ZonMW, registration number: 50–53,000–98–103).”

It should state:

“This study was supported by the Netherlands Organisation for Health Research and Development (ZonMW, project number: 522002013)”.

The author regrets this error.

