# Supplementary material for: Chlamydia trachomatis and the Risk of Pelvic Inflammatory Disease, Ectopic Pregnancy, and Female Infertility: A Retrospective Cohort Study Among Primary Care Patients
Source: Clin Infect Dis. 2019 Aug 24;69(9):1517–25. doi: 10.1093/cid/ciz429 (PMC6792126; doi:10.1093/cid/ciz429)
Supplement: ciz429_suppl_Supplementary_Files [file ciz429_suppl_supplementary_files.docx]

**Supplementary files**

**Full list of confounders**

We considered specific potential confounders for each individual outcome. For PID, potential confounders included a history and/or treatment in the previous month of gonorrhoea, diabetes mellitus, endometriosis and bacterial vaginosis [1], and the use of an intra uterine device (IUD) in the previous 5 years.

For ectopic pregnancy, potential confounders included a history and/or treatment in the previous month of gonorrhoea, diabetes mellitus, (previous) ectopic pregnancy, endometriosis [2], in vitro fertilisation and and the use of an intra uterine device (IUD) in the previous 5 years.

Finally, for the outcome infertility, the following potential confounders were considered: a history and/or treatment in the previous month of gonorrhea [3], diabetes mellitus, polycystic ovary syndrome, primary ovary insufficiency, hyperprolactinemia, endometriosis [2], hypo- and hyperthyroidism, amenorrhea, previous pregnancies, uterine fibroids/myoma’s, amenorrhea [4], autoimmune disorders (such as lupus or rheumatoid arthritis) and the use of folic acid, chemotherapy, immunosuppressive medication [5] and oral contraceptives [6] in the previous 6 months and the use of intra uterine device (IUD) in the previous 5 years [7]. Confounders were included into the final model if they independently changed the β coefficient for the association between CT positive test status and the adverse reproductive outcome by at least 5%.

**Sensitivity analyses**

From 2006 onwards, the British Association for Sexual Health and HIV (BASHH) had no longer recommended Enzyme-Linked Immuno Sorbent Assay (ELISA) for the diagnosis of CT (with a sensitivity of around 40%) and recommended nucleic acid amplification tests (NAAT)s (with sensitivities ranging from 90% to 97%) as the preferred diagnostic lab test [8,9]. In order to assess the impact of this potential change in testing practices, we performed a sensitivity analysis in which the start date of the study was set to 1 January 2007. In addition, because of the impact that contraceptives can have on our outcomes of interest (i.e. ectopic pregnancy and infertility), follow-up time was censored at the time of a contraceptive prescription in a sensitivity analysis.

**References**

1. Brunham RC, Gottlieb SL, Paavonen J. Pelvic inflammatory disease. N Engl J Med, **2015** ; 372: 2039-48.
2. Macer ML, Taylor HS. Endometriosis and infertility: a review of the pathogenesis and treatment of endometriosis-associated infertility. Obstet Gynecol Clin North Am, **2012** ; 39: 535-49.
3. Apari P, de Sousa JD, Müller V. Why sexually transmitted infections tend to cause infertility: an evolutionary hypothesis. PLoS Pathog, **2014** ; 10: e1004111.
4. Meczekalski B, Katulski K, Czyzyk A, Podfigurna-Stopa A, Maciejewska-Jeske M. Functional hypothalamic amenorrhea and its influence on women's health. J Endocrinol Invest, **2014** ; 37: 1049-56.
5. Janssen NM, Genta MS. The effects of immunosuppressive and anti-inflammatory medications on fertility, pregnancy, and lactation. Arch Intern Med, **2000** ; 160: 610-9.
6. Edelman A, Micks E, Gallo MF, Jensen JT, Grimes DA. Continuous or extended cycle vs. cyclic use of combined hormonal contraceptives for contraception. Cochrane Database Syst Rev, **2014** ; 7: CD004695.
7. Hardeman J, Weiss BD. Intrauterine devices: an update. Am Fam Physician, **2014** ; 89: 445-50.
8. Fenton KA, Ward H. National chlamydia screening programme in England: making progress. Sex Trans Infect, **2004** ; 80: 331-3.
9. 2006 UK National Guideline for the Management of Genital Tract Infection with Chlamydia trachomatis, http://www.bashh.org/documents/65.pdf , last accessed 21 December 2018.

**Table S1.** Risk for PID per CT test status when women were censored at the time of a contraceptive prescription

| **Outcome**  **by CT test status** | **N of outcome per category** | **IR**  **(/1000 PYs)** | **Age adjusted HR**  **(95% CI)** | **Adjusted HR**  **(95% CI)** |
| --- | --- | --- | --- | --- |
| **PID** | 3,863 |  |  |  |
| CT untested | 3,702 | 0.7 | 0.71 (0.52 - 0.96) | 0.71 (0.52 - 0.96)^a^ |
| CT negatives | 42 | 1.0 | Reference | Reference |
| CT positives | 119 | 5.8 | 4.16 (2.93 - 5.92) | 3.16 (2.22 - 4.49)^a^ |

Abbreviations: CT, *Chlamydia trachomatis*; IR, incidence rate; HR, hazard ratio; CI, confidence interval; PID, pelvic inflammatory disease; PYs, person years.

^a^ Adjusted for age, smoking and history of gonorrhoea.

**Table S2.** Risk for PID per CT test status when the start date of the study was set at 1 January 2007^a^

| **Outcome**  **by CT test status** | **N of outcome per category** | **IR**  **(/1000 PYs)** | **Age adjusted HR**  **(95% CI)** | **Adjusted HR**  **(95% CI)** |
| --- | --- | --- | --- | --- |
| **PID** | 2,978 |  |  |  |
| CT untested | 2,584 | 0.7 | 0.66 (0.57 - 0.76) | 0.65 (0.57 - 0.75) ^b^ |
| CT negatives | 209 | 1.3 | Reference | Reference |
| CT positives | 185 | 3.5 | 2.66 (2.18 - 3.24) | 2.30 (1.88 - 2.80) ^b^ |

Abbreviations: CT, Chlamydia trachomatis; IR, incidence rate; HR, hazard ratio; CI, confidence interval; PID, pelvic inflammatory disease; PYs, person years.

^a^ Introduction of nucleic acid amplification tests (NAAT) for chlamydia testing in the United Kingdom.^b^ Adjusted for age, smoking and history of gonorrhoea.

**Table S3.** Risk for PID per CT test status, stratified by age categories.

| **Outcome**  **by CT test status** | **N of outcome per category** | **IR**  **(/1000 PYs)** | **Age adjusted HR**  **(95% CI)** | **Adjusted HR**  **(95% CI)** |
| --- | --- | --- | --- | --- |
| **PID** | 8,371^a^ |  |  |  |
| **11-17 years** |  |  |  |  |
| CT untested | 1,036 | 0.4 | 0.43 (0.26 - 0.70) | 0.39 (0.24 - 0.63)^b^ |
| CT negatives | 17 | 1.7 | Reference | Reference |
| CT positives | 27 | 9.7 | 5.29 (2.88 - 9.72) | 3.36 (1.83 - 6.19) ^b^ |
| **18-24 years** |  |  |  |  |
| CT untested | 5,219 | 1.8 | 0.78 (0.67 - 0.91) | 0.75 (0.64 - 0.87) ^b^ |
| CT negatives | 172 | 1.6 | Reference | Reference |
| CT positives | 256 | 6.5 | 3.42 (2.82 - 4.16) | 2.79 (2.30 - 3.39) ^b^ |
| **25-27 years** |  |  |  |  |
| CT untested | 983 | 1.4 | 0.97 (0.68 - 1.40) | 0.98 (0.69 - 1.41) ^b^ |
| CT negatives | 32 | 0.9 | Reference | Reference |
| CT positives | 64 | 4.4 | 3.76 (2.45 - 5.77) | 3.41 (2.22 - 5.24) ^b^ |
| **28+ years** |  |  |  |  |
| CT untested | 518 | 0.8 | 0.59 (0.35 - 0.99) | 0.61 (0.36 - 1.02) ^b^ |
| CT negatives | 15 | 1.2 | Reference | Reference |
| CT positives | 32 | 2.6 | 2.01 (1.09 - 3.72) | 1.95 (1.05 - 3.60) ^b^ |

Abbreviations: PID, pelvic inflammatory disease; CT, Chlamydia trachomatis; IR, incidence rate; HR, hazard ratio; CI, confidence interval; PYs, person years.

^a^ Includes tuberculous PID which resulted in 23 extra CT untested, 1 extra CT negative and 1 extra CT positive cases compared with main analyses (Table 2-4). The percentage of these extra cases on the total number of PID cases per CT test category were: 0.29% (23/7,756) for CT untested, 0.42% (1/236) for CT negatives and 0.26% (1/379) for CT positives.

^b^ Adjusted for age, smoking and history of gonorrhoea.
